# Holmium Laser Enucleation of the Prostate (HoLEP) Versus Transurethral Resection of the Prostate (TURP) in Elderly Patients: Insights Into Recovery, Complications, and Risk Factors

**DOI:** 10.7759/cureus.76384

**Published:** 2024-12-25

**Authors:** Necmi Bayraktar, Ali Barbaros Başeskioğlu

**Affiliations:** 1 Urology, Dr. Burhan Nalbantoğlu State Hospital, Nicosia, CYP; 2 Urology, Cyprus International University School of Medicine, Nicosia, CYP; 3 Urology, Private Practice, Eskişehir, TUR

**Keywords:** benign prostatic hyperplasia (bph), bmi and surgical outcomes, geriatric urology, holmium laser enucleation of the prostate (holep), minimally invasive urology, perioperative management, postoperative complications, prostate surgery outcomes, transurethral resection of the prostate (turp)

## Abstract

Background: We compared the safety and efficacy of holmium laser enucleation of the prostate (HoLEP) and transurethral resection of the prostate (TURP) in elderly men (aged ≥75 years) with benign prostatic hyperplasia (BPH).

Methods: A retrospective analysis of 151 patients (HoLEP: 72; TURP: 79) was conducted. Preoperative and postoperative parameters, including prostate size, International Prostate Symptom Score (IPSS), catheterization duration, hospital stay, and perioperative complications (incontinence and dysuria), were analyzed.

Results: HoLEP significantly reduced catheterization (22 hours vs. 50 hours) and hospitalization times (one day vs. three days) compared to TURP (p < 0.01). However, HoLEP was associated with longer operation times (81.89 min vs. 67.95 min; p < 0.01) and higher rates of dysuria (65.3% vs. 27.8%) and transient incontinence (27.8% vs. 8.9%; p < 0.001).

Conclusion: HoLEP offers significant perioperative benefits over TURP, particularly in shortening recovery times; however, further investigation is required to address the higher rates of dysuria and incontinence. Although the retrospective design and surgeon experience constitute limitations, these findings underscore the need for prospective studies. The results support personalized surgical decision-making, emphasizing patient-specific factors such as body mass index (BMI). These insights may help refine perioperative management and improve collaborative decision-making to enhance outcomes in elderly patients with BPH.

## Introduction

Lower urinary tract symptoms (LUTS) caused by benign prostatic hyperplasia (BPH) are a major health concern, particularly among elderly men. The prevalence of BPH increases with age, affecting up to 90% of men over 80 years old. Given the aging global population, identifying effective and safe surgical interventions for BPH in older adults is critical [1-3].

Transurethral resection of the prostate (TURP) has long been considered the gold standard treatment for BPH, particularly for prostates weighing <80 g. However, TURP carries significant risks including bleeding, transurethral resection syndrome, and prolonged recovery. These challenges have led to the emergence of minimally invasive alternatives such as holmium laser enucleation of the prostate (HoLEP). HoLEP offers advantages such as reduced hospital stay and minimal blood loss; however, it requires advanced surgical expertise and is associated with a steep learning curve.

Several studies have demonstrated the efficacy of HoLEP in reducing hospital stays and perioperative complications compared to TURP, particularly for large prostates (>80 g) [4]. However, limited research has focused specifically on elderly patients (≥75 years), who often present with unique challenges due to comorbidities and age-related physiological changes. Furthermore, the impact of the HoLEP learning curve on outcomes in this demographic population remains underexplored.

The findings of this study have several significant clinical implications. By identifying the benefits and drawbacks of HoLEP versus TURP in elderly patients, we aim to assist urologists in making evidence-based decisions regarding this vulnerable demographic. Additionally, understanding the influence of surgical experience on outcomes can inform training programs and improve procedural safety.

This study sought to address these gaps by comparing the safety and efficacy of HoLEP and TURP in men aged ≥75 years, focusing on perioperative and postoperative outcomes.

## Materials and methods

Study design

This retrospective observational study included 151 patients aged ≥75 years who underwent HoLEP or TURP for BPH between April 2021 and December 2023. All procedures were performed at Dr. Burhan Nalbantoğlu State Hospital, Nicosia, Turkish Republic of Northern Cyprus (TRNC).

Eligibility criteria

Extensive patient evaluation was performed through comprehensive anamnesis, preoperative assessment, complete urinalysis, prostate-specific antigen (PSA) measurement, renal function assessment, fasting blood glucose levels, and documentation of any known systemic diseases. Additionally, prostate size measurements via transabdominal ultrasound and uroflowmetry tests were performed for every patient. The preoperative and postoperative International Prostate Symptom Score (IPSS) were also recorded.

Our exclusion criteria included patients who were not registered or did not follow up, patients with neurogenic disorders, patients with bladder tumors or bladder stones, patients with prostate size <120 ml, patients with prostate size >60 ml, and patients with prostate cancer. The rationale for selecting a specific range of prostate size is to ensure the applicability of both surgical methods (HoLEP and TURP) and to compare the advantages and disadvantages of the methods under equivalent conditions. This range aims to remain within the parameters within which surgical techniques can be applied efficiently and safely and has contributed to enhancing the reliability and comparability of the results.

Data analysis

The statistical tests used in this study were selected based on the nature of the variables. For continuous variables, conformity to normal distribution was tested, and an independent t-test was used to compare the difference between independent groups for data that fit the normal distribution (e.g., operation time and maximum flow rate). The Mann-Whitney U test was preferred for data that did not fit the normal distribution (e.g., duration of catheterization and length of hospital stay). For categorical data, the chi-squared test and Fisher's exact test were used to evaluate the differences between the groups (e.g., postoperative complications and dysuria and incontinence rates). A p-value of less than 0.05 indicated a statistically significant difference. The data analysis was performed using the IBM SPSS Statistics for Windows, V. 29.0 (IBM Corp., Armonk, NY, USA).

Ethical considerations

Patients were informed about the surgical procedure, and written informed consent forms were obtained before starting treatment. This study adhered to the ethical guidelines established by the Declaration of Helsinki and was approved by the Ethics Committee of the Turkish Republic of Northern Cyprus (TRNC) Dr. Burhan Nalbantoğlu State Hospital (project code: 21/24; approval number: YTK.1.01).

Treatment and follow-up protocol

Patients undergoing transurethral procedures were operated on under epidural anesthesia in the lithotomy position by the same surgeon using a rotatable continuous-flow 26-French resectoscope (Olympus, Hamburg, Germany). The irrigation flow rate was set to 60 cm of water, and 0.9% NaCl was used as the irrigation solution. The Olympus ESG-400™ electrosurgical unit (Hamburg, Germany) was employed in patients undergoing TURP surgery, with SalineCut 200-watt effect 2 and SalineCoag 120-watt effect 2 settings (Olympus, Hamburg, Germany). In performing HoLEP, a 100-watt Quantalaser™ (Quanta System, Samarate, Italy) has been utilized in patients. For laser enucleation, 100 watts, 1.2 joules, and 50 hertz virtual basket settings were employed, while 42 watts, 1.2 joules, and 40 hertz bubble blast settings were used for coagulation. The Nesbit technique is used in TURP, while early apical release and en bloc prostatectomy are used in HoLEP [5,6].

## Results

Patient characteristics

A total of 151 patients (TURP: 79, HoLEP: 72) with a mean age of 80.3 ± 4.2 years and a mean prostate volume of 82 ± 20.67 ml were included. While there were no significant differences between the groups in terms of preoperative BMI, IPSS, Qmax, or PSA levels, the HoLEP group had slightly larger prostate volumes than the TURP group (p = 0.014). The patient characteristics are summarized in Table 1.

**Table 1:** Descriptive statistics of patient characteristics by treatment group (HoLEP vs. TURP) *Mean ± SD: data are presented as mean ± standard deviation for normally distributed variables. **Median (IQR): data are presented as median (interquartile range) for non-normally distributed variables. BMI: body mass index (kg/m²); prostate volume: measured in milliliters (ml); IPSS: International Prostate Symptom Score; PVR: post-void residual (ml); Qmax: maximum flow rate (ml/s); PSA: prostate-specific antigen (ng/ml); HoLEP: holmium laser enucleation of the prostate; TURP: transurethral resection of the prostate

Variable	TURP (n = 72)	HoLEP (n = 79)	P-value
Age (years)*	81.09 ± 3.88	79.64 ± 4.61	0.039
BMI (kg/m²)**	26.32 (3.37)	27.35 (3.46)	0.284
Prostate volume (ml)**	75.00 (29.00)	82.00 (43.00)	0.014
IPSS*	23.20 ± 2.62	23.31 ± 2.57	0.80
PVR (ml)			0.38
With catheter	16 (20.3%)	19 (26.4%)	
Less than 100 ml	50 (63.3%)	43 (59.7%)	
More than 100 ml	13 (16.5%)	10 (13.9%)	
Qmax (ml/s)*	10.63 ± 1.85	11.08 ± 1.79	0.13
PSA (ng/ml)**	3.90 (2.63)	3.18 (2.68)	0.25

Perioperative outcomes

Operation Time

HoLEP procedures had significantly longer operation times than TURP (81.89 ± 19.48 min vs. 67.95 ± 11.71 min; p = 0.01; chi-squared test).

Catheterization Time

Median catheterization time was significantly shorter for HoLEP (22 hours vs. 50 hours; p = 0.01; chi-squared test).

Hospitalization

**Figure 1:**
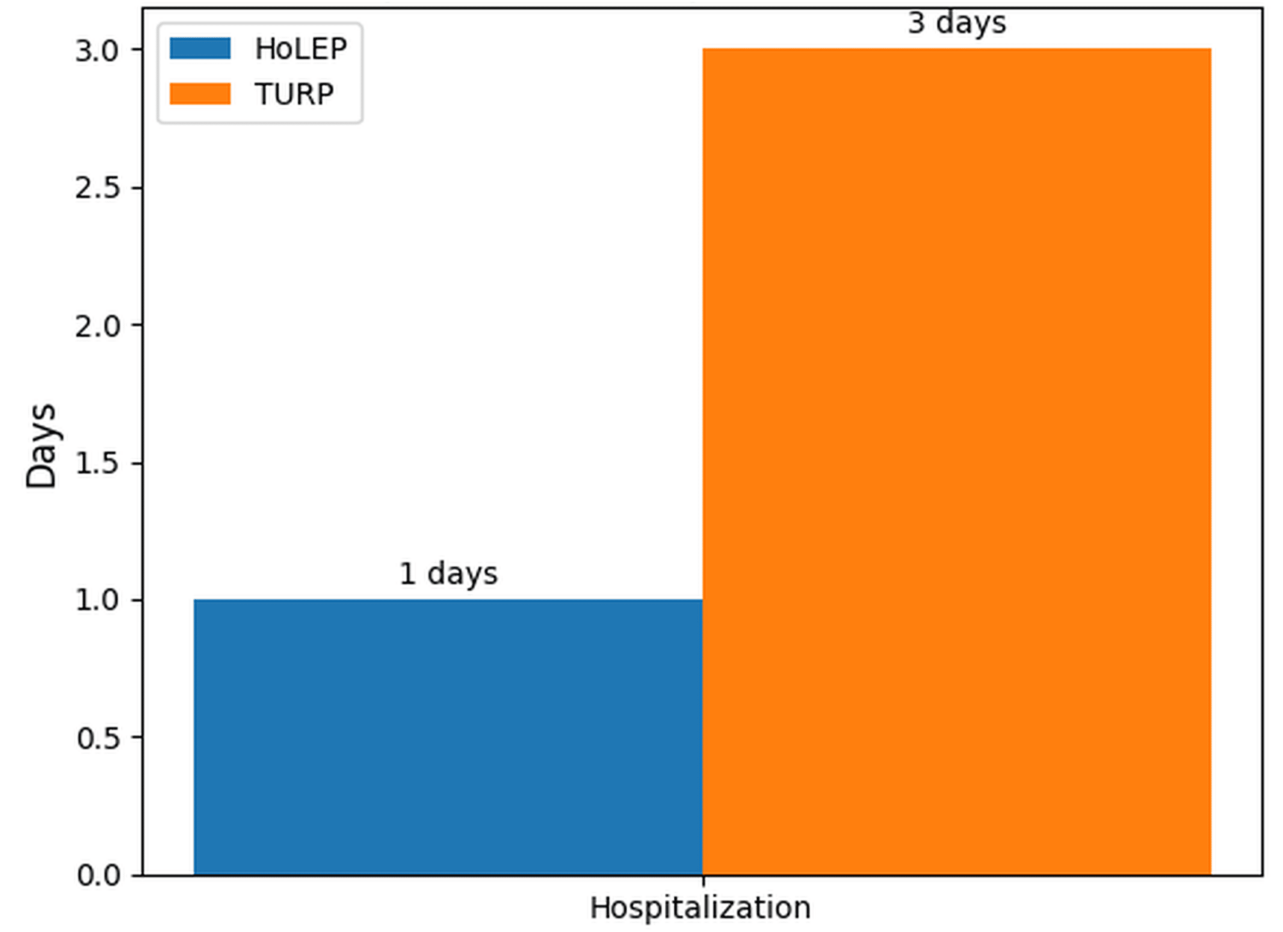
Comparison of hospitalization duration HoLEP: holmium laser enucleation of the prostate; TURP: transurethral resection of the prostate

HoLEP patients had a shorter hospital stay than TURP patients (one day vs. three days; p = 0.01; chi-squared test) (Figure 1).

Irrigation Needs

The HoLEP group required significantly less irrigation time and fluid (p = 0.01; chi-squared test).

The mean operation time was significantly longer in the HoLEP group (81.89 ± 19.48 min) than in the TURP group (67.95 ± 11.71 min) (p < 0.001; chi-squared test). The effect size measured by Cohen's d was 0.877, indicating a medium-to-large effect size. This indicates that the difference in the operation time was statistically significant and clinically meaningful. Hedges' correction, which accounts for a small sample size bias, was 0.873, supporting the result of a significant difference.

The median irrigation time was significantly shorter in the HoLEP group (6.00 hours, IQR 3.00) than in the TURP group (23.00 hours, IQR 5.00) (p < 0.001). Irrigation fluid consumption was also significantly lower in the HoLEP group; median values were 5.00 L (IQR 1.00) for HoLEP and 16.00 L (IQR 5.00) for TURP (p < 0.001). The median catheterization time was significantly shorter in the HoLEP group (22.00 days, IQR 6.00) than in the TURP group (50.00 days, IQR 9.00) (p < 0.001). The median hospital stay of patients in the HoLEP group (1.00 days, IQR 0.00) was significantly shorter than that of patients in the TURP group (3.00 days, IQR 1.00) (p < 0.001). Perioperative and postoperative findings are presented in Table 2.

**Table 2:** Perioperative and postoperative findings by treatment group (HoLEP vs. TURP) Values are presented as mean ± SD or median (IQR) based on the normality of the data distribution. An independent t-test was used for normally distributed variables, and the Mann-Whitney U test was used for non-normally distributed variables. SD: standard deviation; IQR: interquartile range; L: liter; HoLEP: holmium laser enucleation of the prostate; TURP: transurethral resection of the prostate

	TURP (n = 72)	HoLEP (n = 79)	P-value
Operation time (minutes)* mean ± SD	67.95 ± 11.71	81.89 ± 19.48	<0.01
Irrigation time (hours)** median (IQR)	23.00 (5.00)	6.00 (3.00)	<0.01
Irrigation liquid consumed (L)** median (IQR)	16.00 (5.00)	5.00 (1.00)	<0.01
Catheterization time (hours)** median (IQR)	50.00 (9.00)	22.00 (6.00)	<0.01
Hospitalization time (days)** median (IQR)	3.00 (1.00)	1.00 (0.00)	<0.01

Postoperative complications

Dysuria

**Figure 2:**
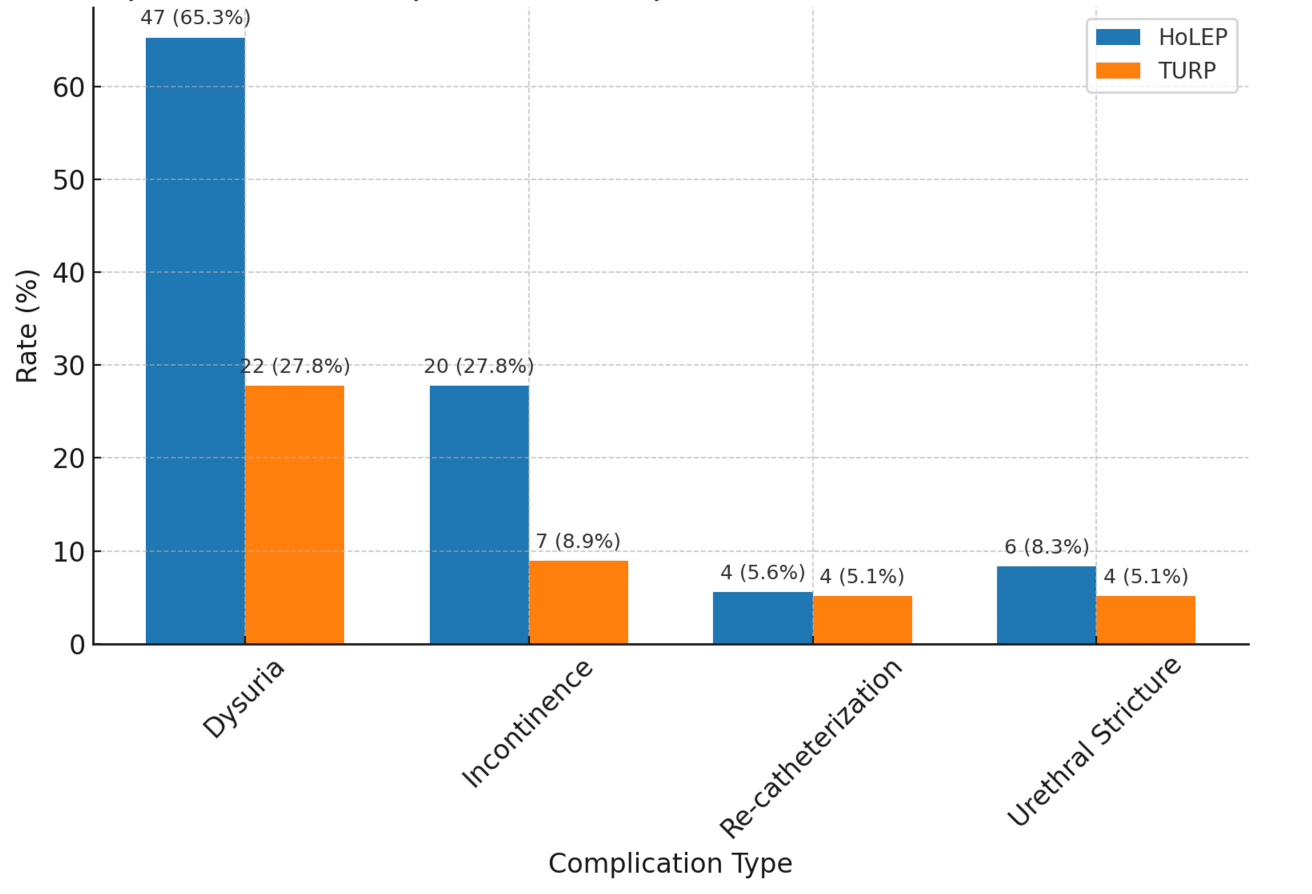
Comparison of postoperative complications between the HoLEP and TURP groups HoLEP: holmium laser enucleation of the prostate; TURP: transurethral resection of the prostate

Postoperative dysuria was observed in 65.3% of HoLEP patients compared with 27.8% of TURP patients (p < 0.001; chi-squared test), as shown in Figure 2.

Incontinence

Transient incontinence was significantly higher in the HoLEP group (27.8%) than in the TURP group (8.9%; p < 0.001; chi-squared test).

Re-catheterization and Stricture Rates

No significant differences were noted between the groups for re-catheterization (HoLEP, 5.6%; TURP, 5.1%; p = 0.188) or urethral stricture (HoLEP, 8.3%; TURP, 5.1%; p = 0.421; chi-squared test).

Functional outcomes at the four-month follow-up

IPSS Improvement

Both groups showed significant improvement in IPSS scores, with no statistically significant difference between them (HoLEP: 12.85 ± 1.84 vs. TURP: 13.44 ± 2.02; p = 0.055).

Qmax

Maximum urine flow rates were comparable between the groups at four months (HoLEP: 23.07 ± 3.11 ml/s vs. TURP: 24.01 ± 4.27 ml/s; p = 0.126).

The confidence intervals were 12.41-13.28 for the HoLEP group and 12.98-13.89 for the TURP group, suggesting that the difference between the procedures may be minimal and not clinically significant. Confidence intervals were 22.34-23.80 ml/s in the HoLEP group and 23.01-25.01 ml/s for the TURP group, respectively, indicating that the difference between the procedures may be minimal and not clinically significant. The incidence of dysuria during the postoperative follow-up was significantly higher in the HoLEP group than in the TURP group (HoLEP, 65.3%; TURP, 27.8%; p < 0.001). The urethral stricture rates were comparable in both groups (HoLEP, 8.3%; TURP, 5.1%; p = 0.421). The postoperative urinary incontinence rate was significantly higher in the HoLEP group than that in the TURP group (HoLEP, 27.8%; TURP, 8.9%; p < 0.001). No statistically significant difference was observed in the postoperative prostate cancer detection rates (HoLEP, 4.2%; TURP, 5.1%; p = 0.302). There was no significant difference in the need for re-catheterization due to bleeding or inability to urinate between the groups (HoLEP, 5.6%; TURP, 5.1%; p = 0.188). In the transabdominal ultrasound control performed six weeks after surgery, the median residual prostate tissue was 33 (13) in the TURP group and 26 (eight) in the HoLEP group, with a statistically significant difference between the two groups (p = 0.049). Postoperative results are shown in Table 3.

**Table 3:** Comparison of postoperative outcomes between the HoLEP and TURP groups ^a^Mann-Whitney U test. ^b^Independent t-test. ^c^Postoperative residual prostate (transabdominal ultrasound measurement). ^d^Chi-squared test. N: indicates the number of patients in each group. Mean ± SD: indicates the mean value and standard deviation. p-value: statistical significance. HoLEP: holmium laser enucleation of the prostate; TURP: transurethral resection of the prostate; IQR: interquartile range

Outcome measure	HoLEP group (n = 72)	TURP group (n = 79)	P-value
Four-month follow-up IPSS^a^ mean ± SD	12.85 ± 1.84	13.44 ± 2.02	0.055
Four-month follow-up Qmax (ml/s)^ b^ mean ± SD	23.07 ± 3.11	24.01 ± 4.27	0.126
Postoperative residual prostate^c^ median (IQR)	26 (8)	33 (13)	0.049
Postoperative dysuria^d^	47 (65.3%)	22 (27.8%)	<0.001
Re-catheterization^d^	4 (5.6%)	4 (5.1%)	0.188
Postoperative incontinence^d^	20 (27.8%)	7 (8.9%)	<0.001
Urethral stricture^d^	6 (8.3%)	4 (5.1%)	0.421
Prostate cancer detection^d^	3 (4.2%)	4 (5.1%)	0.302

Postoperative dysuria and incontinence were observed to be significantly higher in the HoLEP group. In patients with an elevated BMI (BMI >30 kg/m²), incontinence rates increased in the HoLEP group. Chi-squared analysis revealed a significant association between the HoLEP procedure and incontinence in patients with a BMI >30 kg/m² (p = 0.007).

Statistical analysis of confounding factors

Logistic regression analysis indicated that BMI significantly influenced transient incontinence rates (odds ratio (OR) = 57.27; 95% CI: 13.725-238.990; p < 0.001). However, when BMI was controlled, the surgical group (HoLEP vs. TURP) was not a significant predictor of incontinence (p = 0.403). However, when binary logistic regression analysis evaluated BMI and surgical group (TURP vs. HoLEP) variables concurrently, although elevated BMI was identified as a significant risk factor (OR = 57.27; 95% CI: 13.725-238.990; p < 0.001), the group effect (TURP vs. HoLEP) was not statistically significant (p = 0.403).

Logistic regression analysis of BMI categorical data revealed an OR (Exp(B)) of 32.129, a 95% CI of 8.014, 128.810, and a p-value of <0.001. This outcome indicates that individuals in the high BMI category had a 32-fold greater probability of experiencing incontinence than those in the low BMI group. These findings were statistically significant (p < 0.001). A chi-squared analysis for catheterization duration yielded a p-value of 0.073, which was close to statistical significance (p = 0.05). Nonetheless, binary logistic regression analysis produced an OR for catheterization duration (Exp(B)) of 0.982, a 95% CI of 0.950, 1.014, and a p-value of 0.263. The proximity of this OR to 1 suggests that this variable does not substantially influence incontinence development.

According to multiple logistic regression analysis, preoperative prostate volume (p = 0.983), IPSS (p = 0.718), post-void residual (PVR) (p = 0.792), PSA (p = 0.342), duration of operation (p = 0.879), irrigation duration (p = 0.133), and irrigation quantity (p = 0.876) had no significant effect on postoperative incontinence (p > 0.05). Similarly, no variable was found to have a significant effect on the occurrence of postoperative dysuria. Factors such as irrigation time, amount of irrigation spent, and prostate volume may be clinically important but were not found to be statistically significant in our study.

## Discussion

TURP has long been recognized as the "gold standard" surgical option in the treatment of BPH. However, the inadequacy of tissue resection in large prostates and well-known complications associated with this method have led to the search for alternative surgical approaches such as HoLEP [7]. A significant contribution of this study lies in its comparative analysis of HoLEP and TURP in elderly patients aged >75 years. Although numerous studies comparing HoLEP and TURP exist in the literature, research focusing on the elderly population remains limited. Although similar findings have been reported in the literature, this study presents novel insights derived from real-world data in the elderly population.

In the HoLEP group, the longer observed operation time may be attributable to the more technically complex nature of the procedure than that of TURP. Numerous studies focusing on HoLEP emphasize that with increased surgical experience and a greater number of cases, operation times significantly decrease. This observation aligns with previous research, which suggests that HoLEP operative time decreases as surgeons progress along the learning curve. Recent studies have demonstrated a trend of decreased operative times as surgeons gain proficiency in HoLEP, particularly in cases involving larger prostates [8-10]. This supports the notion that with sufficient experience, HoLEP can serve as a viable alternative to TURP, especially for managing large prostate volumes, while maintaining lower perioperative complications and improved postoperative outcomes.

The observed reduction in irrigation time and volume in the HoLEP group suggests that patients undergoing this procedure may experience a quicker recovery than those undergoing TURP. This may be due to less tissue damage and the precision of the HoLEP laser-based technique. Studies indicate that HoLEP minimizes blood loss and reduces the need for prolonged irrigation, which aligns with existing research emphasizing its ability to achieve better hemostasis and lower perioperative complications, particularly in larger prostates.

The significant reduction in irrigation time and fluid volume in the HoLEP group suggests faster recovery, likely due to reduced tissue trauma and the precision of the laser-based technique. Although the available data do not yet allow firm conclusions to be drawn, evidence suggests that HoLEP is effective in minimizing blood loss and reducing the need for irrigation. For example, a study on bladder irrigation following HoLEP found no increase in postoperative complications such as bleeding or urinary retention despite the absence of prolonged irrigation, emphasizing the efficacy of HoLEP in providing hemostasis and limiting the need for postoperative irrigation [11]. Another study showed that HoLEP significantly reduced intraoperative blood loss, which was associated with a reduced need for intraoperative and postoperative irrigation [12]. These findings align with the broader literature that emphasizes the efficacy of HoLEP in managing perioperative outcomes, particularly in patients with large prostate volumes.

In our study, a significantly shorter catheterization time in HoLEP patients may mean lower discomfort, fewer catheter-related complications, and faster recovery. Several recent studies support this observation, showing that HoLEP patients tend to have shorter catheterization times and earlier hospital discharge than TURP patients, leading to reduced hospitalization costs [13,14]. One study noted that a shorter catheterization time in HoLEP directly affects hospital costs and postoperative morbidity [15]. This trend is in line with the finding that a faster recovery is associated with fewer perioperative complications [7].

In our study, there was no significant difference in the clinical efficacy between the treatment groups. However, a surgeon's level of experience plays a critical role in influencing treatment outcomes. The surgeon had more than 15 years of experience with TURP, but only three years with HoLEP, potentially leading to bias. Studies have shown that as the surgeon becomes more experienced and proficient in this technique, the results of HoLEP improve [13]. The learning curve for HoLEP is well documented, and it is likely that with further exposure and skill acquisition, the difference between HoLEP and TURP outcomes diminishes [16,17]. Future studies should consider the surgeon's learning curve when evaluating procedural outcomes, as increased experience can significantly impact perioperative efficiency, complication rates, and patient recovery times [17,18]. 

In our investigation, no single variable had a statistically significant effect on postoperative dysuria. Although factors such as irrigation time, irrigation volume, and prostate volume may be clinically relevant, they were not statistically significant. These findings are consistent with recent literature suggesting that variables such as prostate size and surgical technique influence postoperative recovery, although larger datasets are often necessary to detect more subtle effects [19]. Nevertheless, despite the statistically insignificant results for certain factors, we observed a significantly higher incidence of postoperative dysuria in the HoLEP group than in the TURP group (HoLEP: 65.3% vs. TURP: 27.8%; p < 0.001). This observation suggests that the HoLEP procedure may induce increased irritation or inflammation, which is potentially associated with more extensive tissue resection. The observed dysuria may represent a transient effect that diminishes as healing progresses. Future studies involving larger, more diverse patient groups are needed to determine whether these findings are generalizable and the precise mechanisms underlying the increased dysuria observed in patients with HoLEP [20].

The rate of postoperative urinary incontinence was significantly higher in the HoLEP group than in the TURP group (HoLEP, 27.8%; TURP, 8.9%; p < 0.001). This disparity may be attributed to the more invasive nature of the HoLEP procedure or the surgeon's position on the learning curve, as HoLEP involves more extensive tissue removal than TURP. However, numerous studies have demonstrated that as surgical experience increases, the rate of transient urinary incontinence, often considered an inherent disadvantage of the HoLEP procedure, significantly decreases over time [21,22]. As surgeons become proficient in HoLEP, the duration and severity of incontinence diminish, indicating that this complication may be closely associated with the learning curve of the procedure rather than a permanent disadvantage. Furthermore, recent evidence suggests that the risk of transient incontinence after HoLEP is manageable and continues to decrease with increasing expertise, rendering the technique an increasingly viable option even in larger prostate cases [23]. This underscores the importance of surgical experience and patient selection in reducing postoperative complications and reinforces the need for continuous skill development in HoLEP procedures.

In our study, patients with a BMI >30 kg/m² appeared to benefit more from TURP than from HoLEP. Although more robust statistical evidence is lacking in this regard, this observation warrants further investigation in large-scale studies to critically assess these findings. Additionally, given that prostate size was limited to cases suitable for TURP or near the upper limit of effective resection, the generalizability of our comparison to all patients with BPH is limited. Therefore, the conclusions drawn from this study may not apply to patients with significantly larger prostate volumes, and further research is needed to comprehensively explore these aspects in more diverse patient populations.

It is also crucial to consider that the differing resection techniques and learning curves associated with HoLEP and TURP may have influenced the outcomes observed in this study. Variations in patient characteristics, such as BMI, may interact with these surgical approaches differently, further highlighting the need for future studies to include a wider range of prostate sizes and patient demographics to enhance the generalizability of the findings.

This study has several limitations. The primary limitation of this study is its retrospective design, which may introduce selection bias and limit the causal interpretation of results. Additionally, the sample size remains relatively small for broader generalization. Given that the surgeon's experience with HoLEP was limited, less favorable outcomes may have been obtained with this method. Considering the learning curve associated with HoLEP, further studies involving experienced surgeons would yield more reliable results. Factors such as BMI and prostate volume may also influence the results; therefore, additional studies with larger populations are warranted. Moreover, randomization was not implemented, and patients were consecutively included. While this approach does not offer the advantage of bias reduction provided by randomized trials, it reflects real-world data.

## Conclusions

This study shows that HoLEP offers significant perioperative benefits, including shorter catheterization and hospitalization times, compared to TURP in elderly patients with BPH. However, higher rates of transient dysuria and incontinence with HoLEP highlight the need for improved perioperative management and surgeon expertise. BMI was identified as a risk factor for incontinence, emphasizing the importance of patient-specific factors in surgical planning. While HoLEP is a promising alternative to TURP, further prospective studies are required to validate these findings and address postoperative complications.
